# Riboflavin Increases Goat Sperm Motility via Enhancement of Mitochondrial β-Oxidation

**DOI:** 10.3390/biology15010085

**Published:** 2025-12-31

**Authors:** Qian Wang, Nan Zhang, Linlin Sun, Pigang Ding, Shengyan Zhao, Dongping Ma, Xin Kou, Zhendong Zhu, Lingjiang Min

**Affiliations:** 1 College of Animal Science and Technology, Qingdao Agricultural University, Qingdao 266109, China; 13673940033@163.com (Q.W.); 18853508831@163.com (L.S.); zzd2020@qau.edu.cn (Z.Z.); 2 Qinghai Conservation and Utilization Center of Livestock and Poultry Genetic Resources, Xining 810000, China; 353127579@163.com; 3 Yifengdian Town Animal Health and Product Quality Supervision Station, Jimo District, Qingdao 266224, China; d564969662@163.com; 4 Shouguang City Animal Husbandry Development Center, Weifang 262700, China; sdsgzsy@163.com (S.Z.); sgylmdp@163.com (D.M.); 5 Hongde Livestock Farm, Yingli Town, Weifang 262700, China; kouxin2004@126.com

**Keywords:** goat, mitochondrial, metabolism, riboflavin, sperm quality, β-oxidation

## Abstract

Mitochondrial energy metabolism is fundamental to sperm function, and fatty acid β-oxidation is an important pathway for adenosine triphosphate (ATP) production. Riboflavin, a precursor of key flavin cofactors, plays a critical role in regulating β-oxidation and supports multiple physiological processes. This study aimed to determine whether adding riboflavin to semen dilution media could enhance goat sperm motility and to elucidate the underlying metabolic mechanisms. Goat semen was diluted in tris-citrate-glucose (TCG) medium containing 0, 5, 10, 15, and 20 μM riboflavin and incubated at 37 °C, after which sperm motility, acrosome integrity, mitochondrial membrane potential, ATP levels, malate dehydrogenase (MDH) and succinate dehydrogenase (SDH) activities, and the NADH/NAD^+^ were evaluated. The localization and expression of the β-oxidation enzymes carnitine palmitoyltransferase 1 (CPT1) and extremely long chain acyl-CoA dehydrogenase (ACADVL) were examined, and CPT1 activity was quantified. The results showed that CPT1 and ACADVL were present in goat sperm, and that 10 μM riboflavin significantly increased sperm motility, acrosome integrity, mitochondrial activity, ATP levels, and the activities of MDH, SDH, and CPT1, while also elevating NADH/NAD^+^ levels (*p* < 0.05). Notably, these enhancements were suppressed by 100 μM etomoxir, a mitochondrial β-oxidation inhibitor, which reduced total motility, ATP Levels, and CPT1 activity after riboflavin supplementation (*p* < 0.05). These findings indicate that goat sperm at least partly rely on mitochondrial β-oxidation for ATP generation and that riboflavin supplementation enhances mitochondrial metabolism, thereby improving sperm quality.

## Simple Summary

Artificial insemination (AI) is a widely used assisted breeding technique in goats, and improving sperm quality is key to enhancing AI efficiency. Sperm motility and function depend on a continuous supply of energy, and the fatty acid β-oxidation pathway regulated by riboflavin-derived cofactors is one of the energy sources for sperm. In this study, goat semen was treated with different concentrations of riboflavin, and sperm quality was assessed after incubation. Riboflavin supplementation significantly increased sperm motility, ATP levels, and the activity of key metabolic enzymes, whereas the addition of β-oxidation inhibitors reversed these improvements. These results indicate that riboflavin enhances sperm energy metabolism through the fatty acid β-oxidation pathway, thereby improving overall sperm quality. This finding could help increase the success rate of AI in goats, improve reproductive efficiency, and provide economic benefits for animal production.

## 1. Introduction

Energy metabolism constitutes the fundamental driving force that supports sperm physiological function [1]. During mammalian fertilization, sperm must traverse the complex microenvironment of the female reproductive tract, a process that involves several physiological events—including capacitation, the acrosome reaction, and the zona pellucida interaction—each of which requires a continuous supply of energy. Adenosine triphosphate (ATP), the primary cellular energy currency, plays a pivotal role in regulating flagellar motility, ion channel activity, and intracellular signaling cascades in sperm [2,3]. In mammalian sperm, ATP is synthesized via both glycolysis, which occurs in the fibrous sheath of the principal flagellar piece, and oxidative phosphorylation (OXPHOS) in the mitochondrial midpiece [4]. The unique helical sheath-like arrangement of mitochondria in the midpiece is thought to enhance energy transduction efficiency [5]. A previous study demonstrated that goat sperm maintain basal ATP levels through mitochondrial respiratory chain activity even when glycolysis is pharmacologically inhibited by 3-chloro-1,2-propanediol (3-MCPD) [6], indicating that OXPHOS plays an essential role in ATP generation required for maintaining sperm function.

OXPHOS is a central process in cellular energy metabolism, primarily occurring within the inner mitochondrial membrane [7]. During this process, electron donors transfer high-energy electrons to the electron transport chain (ETC), triggering a series of redox reactions that ultimately drive ATP synthase to catalyze the phosphorylation of ADP into ATP [8]. Among the substrates fueling OXPHOS, fatty acids represent an important energy source. Through fatty acid oxidation (FAO), long-chain fatty acids are progressively degraded via β-oxidation to yield acetyl-CoA, NADH, and FADH_2_ [9,10]. Acetyl-CoA enters the tricarboxylic acid (TCA) cycle, generating additional reducing equivalents, whereas NADH and FADH_2_ donate electrons to the ETC to support efficient ATP production [11,12]. FAO therefore plays a crucial role in maintaining mitochondrial energy supply, regulating lipid balance, and modulating important signaling pathways within cells [13]. This pathway has important functional implications in cell types with high metabolic requirements, especially sperm cells [14]. Flavin mononucleotide (FMN) and flavin adenine dinucleotide (FAD) are indispensable redox cofactors that play a key role in cellular metabolism. FMN and FAD participate directly in electron transfer within the ETC and are required for key metabolic reactions in the TCA cycle, β-oxidation, and amino acid metabolism [15,16]. Adequate availability of these cofactors is critical for maintaining ATP production, mitochondrial function, and redox balance, thereby safeguarding overall cellular health [17]. Therefore, the adequate supply of FMN and FAD is vital for mitochondrial function and overall cellular health.

Riboflavin (vitamin B_2_) is an essential water-soluble micronutrient and the biochemical precursor of the flavin cofactors FAD and FMN [18,19]. Because endogenous riboflavin storage is minimal and largely restricted to hepatic and renal tissues, continuous dietary intake or nutritional supplementation is necessary to meet cellular metabolic demands [20]. Suboptimal dietary riboflavin intake or genetic mutations affecting its metabolic activation pathways can severely disrupt intracellular FAD and FMN synthesis, thereby compromising energy metabolism [21]. Riboflavin deficiency impairs multiple flavoprotein-dependent pathways, including enzymes within the TCA cycle, the fatty acid β-oxidation pathway, and components of the ETC, ultimately reducing ATP production efficiency [22,23]. Beyond its metabolic roles, Riboflavin also contributes significantly to cellular redox homeostasis, exhibiting antioxidant, anti-inflammatory and cytoprotective properties, and has been widely used as a therapeutic supplement in various clinical and nutritional contexts [24]. In the context of reproduction, studies in animal models have shown that riboflavin supplementation alleviates fluoride-induced sperm toxicity, restores motility and membrane integrity, and mitigates testicular damage [25]. Disruption of riboflavin metabolism has also been linked to abnormal testicular development and impaired spermatogenesis, particularly under high-fat diet conditions [26]. Perturbations in FAD biosynthesis during germ cell differentiation can lead to aberrant sperm morphogenesis and reduced sperm quality [27]. Notably, the solute carrier protein SLC22A14 has been identified as a critical mitochondrial riboflavin transporter in mouse sperm, facilitating efficient riboflavin uptake and thereby supporting mitochondrial fatty acid β-oxidation and overall energy homeostasis [28]. Despite these findings, the molecular mechanisms through which riboflavin regulates sperm quality remain incompletely understood. Based on the central role of FAD and FMN in mitochondrial β-oxidation, we hypothesized that riboflavin supplementation may enhance sperm mitochondrial fatty acid oxidation, thereby improving ATP production, maintaining energy homeostasis, and ultimately promoting sperm motility and function. To test this hypothesis, the present study investigates the effects of riboflavin supplementation on goat sperm quality and mitochondrial metabolic activity.

## 2. Materials and Methods

### 2.1. Semen Collection and Processing

Six healthy and fertile dairy goats from Jimo, Qingdao, were used in this study. The goats were kept individually under natural conditions, with unrestricted access to water and a diet of commercial feed. During the November breeding season, semen was collected twice per week using an artificial vagina and promptly transported to the laboratory for evaluation. A total of 48 semen samples were used in this study, and three repeated experiments were conducted. Only those with more than 80% sperm motility were selected for use, and then eligible fresh semen samples were pooled to reduce individual differences and divided equally into five aliquots. Each aliquot was diluted with TCG buffer containing varying concentrations (0, 5, 10, 15, and 20 µM) of riboflavin (R817215, 98%, Macklin, Shanghai, China), adjusting the final sperm concentration to 100 × 10^6^ sperm/mL. The TCG buffer consisted of 250 mM Tris, 83 mM citric acid, and 69 mM D-glucose. Samples were incubated at 37 °C for varying periods (0, 1, 3, and 6 h) before sperm parameters were analyzed. All the sample preparation and incubation steps were carried out under light-proof conditions. To inhibit the mitochondrial β-oxidation process, some other samples were also treated with different concentrations (0, 1, 10, 100, and 1000 µM) of etomoxir (HY-50202, 99.73%, MCE, Monmouth Junction, NJ, USA).

### 2.2. Assessment of Sperm Motility

As described in our previous research [29], sperm motility and kinematic parameters were evaluated using a computer-assisted sperm analysis (CASA) system (HT CASA-Ceros II; Hamilton Thome, MA, USA). A 5 µL portion of the semen sample was loaded into a pre-warmed Makler chamber and examined under 10× magnification. Three randomly selected microscopic fields were analyzed, and sperm cells within these areas were assessed.

### 2.3. Sperm Viability and Acrosome Integrity

Sperm viability and acrosome integrity were assessed using FITC-PNA/PI double staining. According to the protocol outlined in a previous study [30], goat semen samples were first diluted in PBS to a final sperm concentration of 1 × 10^6^ sperm/mL for flow cytometric analysis. Subsequently, 0.54 µL propidium iodide (PI) and 0.6 µL fluorescein isothiocyanate-peanut agglutinin (FITC-PNA) were added for staining. After thorough mixing, samples were tested by incubating at 37 °C for 5 min in the dark. Sperm were analyzed with the use of a flow cytometer (BeamCyte, Changzhou, China), with a 530/30 nm bandpass filter to record the green fluorescence emitted by FITC-PNA and a 585/42 nm bandpass filter to record the red fluorescence emitted by PI. 20,000 events were recorded for each sample (n = 3), and the raw data was analyzed using FlowJo (v10.8.1). FITC-PNA negative sperm were classified as having intact acrosome, whereas those with negative PI staining were viable with intact membranes.

### 2.4. Mitochondrial Membrane Potentials

According to our previous study [31], sperm mitochondrial membrane potential (MMP) was evaluated using the JC-1 MMP Assay Kit (Beyotime Biotechnology, Shanghai, China). In accordance with the manufacturer’s protocol, sperm samples were incubated with JC-1 working solution at 37 °C in the dark for 20 min. Following incubation, the samples were centrifuged at 600× *g*, washed three times with JC-1 buffer, and the resulting sperm pellet was resuspended for flow cytometric analysis. Each analysis was conducted in triplicate (n = 3).

### 2.5. Assessment of Sperm ATP Levels

The ATP levels in sperm were measured using an ATP Content Assay Kit (Nanjing Jiancheng Bioengineering Institute, Nanjing, China). The collected sperm samples were homogenized and disrupted in an ice-water bath, followed by heating in boiling water for 10 min. After thorough mixing, the samples were prepared for analysis. 30 µL aliquot of each sample was sequentially treated with the working solution, chromogenic reagent, and stop solution. The reaction mixtures were then transferred to a 96-well plate, and absorbance was measured at 636 nm using a microplate reader. All assays were performed in triplicate (n = 3).

### 2.6. Assessment of Sperm MDH and SDH Activities

Malate dehydrogenase (MDH) and succinate dehydrogenase (SDH) assay kits (Nanjing Jiancheng Bioengineering Institute, Nanjing, China) were used to measure the activities of MDH and SDH [32]. Sperm were resuspended in PBS as a homogenization buffer and lysed by sonication (20 kHz, 750 W, 40% amplitude). Subsequently, it was centrifuged at 2000× *g* for 10 min at 4 °C to collect the supernatant for subsequent analysis. The resulting supernatant was then transferred into a 96-well plate, where the pre-warmed working solution was added and thoroughly mixed, and the activities of MDH and SDH were assessed using a microplate reader at 340 nm and 600 nm, respectively. All assays were performed in triplicate (n = 3).

### 2.7. Assessment of Sperm CPT1 Activities

Carnitine palmitoyltransferase 1 (CPT1) activity was measured using CPT1 assay kit (Mlbio, Shanghai, China) following the manufacturer’s protocol. In brief, the enzymatic reaction generates acyl-carnitine and it releases free sulfhydryl groups from coenzyme A (CoA-SH), which reacts with the Ellman’s reagent DN-TB (5,5′-dithiobis-2-nitrobenzoic acid) to produce yellow-colored 5-thio-2-nitrobenzoic acid (TNB). The absorbance of TNB was measured at 412 nm, and the rate of absorbance change was used to quantify CPT1 activity. All assays were performed in triplicate (n = 3).

### 2.8. Determination of NADH/NAD^+^ Levels

The levels of NADH and NAD^+^ in sperm were determined using a coenzyme I NAD (H) assay kit (Nanjing Jiancheng Bioengineering Institute, Nanjing, China). NADH and NAD^+^ were extracted separately from the samples using acidic and alkaline extraction buffers. Following manufacturer’s instructions, the working solution was added, and absorbance was measured at 570 nm to quantify their concentrations.

### 2.9. Western Blotting

Total sperm protein was extracted using a lysis buffer. The extracted proteins were separated using 10% SDS-PAGE (EC1024-B, Sparkjade, Jinan, China) and subsequently transferred onto PVDF membranes. Non-specific binding sites were blocked by incubating the membranes in TBST containing 5% bovine serum albumin (BSA) for 2 h at room temperature. Primary antibodies were diluted in 1% BSA and incubated with the membranes overnight at 4 °C. The following primary antibodies were used, CPT1A (Rabbit pAb, 15184-1-AP, 1:10,000; Proteintech, Wuhan, China), ACADVL/VLCAD (HA722416, 1:1000; Hubio, Hangzhou, China), and α-Tubulin (Rabbit mAb, AC049, 1:20,000; ABclonal, Wuhan, China). After washing with TBST, the membranes were incubated for 1 h with an HRP-conjugated goat anti-rabbit secondary antibody (AS014, 1:5000; ABclonal, Wuhan, China). After washing with TBST three times, the protein bands were visualized using the gel imaging system.

### 2.10. Immunofluorescence Staining

As described in previous studies, sperm samples were rinsed with PBS, mounted on poly-L-lysine-coated slides, fixed with methanol for 10 min, and subsequently air-dried at room temperature. At room temperature, non-specific binding was blocked for 30 min with PBST containing 10% BSA. Samples were then incubated with anti-CPT1 (1:200) and anti-ACADVL (1:100) overnight at 4 °C. The following day, after PBS washes, the samples were incubated with biotinylated goat anti-rabbit FITC-conjugated IgG (1:200). After washing, the sperm were counterstained with DAPI (CWBIO). Fluorescence images were acquired using a fluorescence microscope (80i, Nikon, Tokyo, Japan). Negative controls were included by omitting the primary antibody during immunostaining.

### 2.11. Statistical Analysis

Three duplicates of data were compared, multiple comparisons were made using one-way ANOVA, and then Tukey post hoc tests were used. The results were expressed as the means ± standard deviation (SD). When *p* < 0.05, the difference between treatments was considered statistically significant.

## 3. Results

### 3.1. Riboflavin Improved Sperm Motility Parameters During Incubation at 37 °C

As shown in Table 1, after 1 h of incubation, sperm progressive motility was significantly higher in the riboflavin-treated groups than in the control (*p* < 0.05). However, total motility showed no statistical differences. At 3 h, compared with the control group, the addition of riboflavin significantly increased the total motility of sperm (*p* < 0.05). Among them, 5 and 10 μM riboflavin significantly enhanced the progressive motility, curvilinear velocity (VCL), straight-line velocity (VSL), average path velocity (VAP), linearity (LIN), and beat-cross frequency (BCF) of sperm (*p* < 0.05). After incubation for 6 h, the progressive motility of sperm in all riboflavin treatment groups was still significantly higher than that in the control group (*p* < 0.05). It is notable that, except for lateral head displacement (ALH), straightness (STR) and LIN, the 10 μM riboflavin group performed the best in all parameters (*p* < 0.05).

### 3.2. Riboflavin Improved the Sperm Viability and Acrosome Integrity

**Figure 1:**
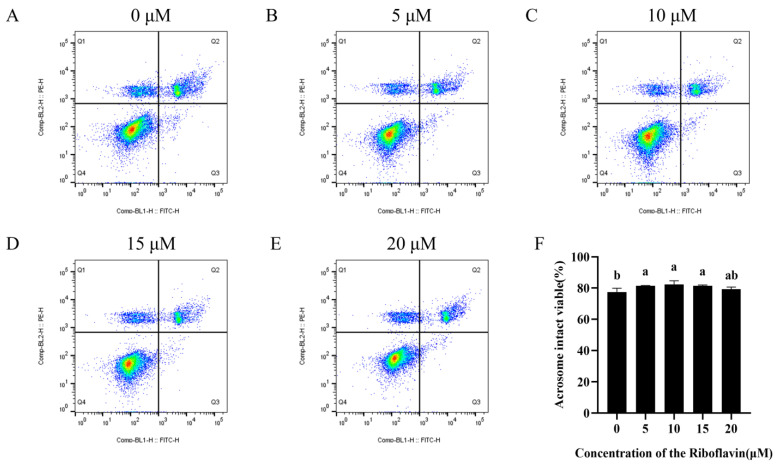
Effect of riboflavin on sperm viability and acrosome integrity in goats after 3 h incubation. (**A**–**E**) Flow cytometric analysis of sperm vitality and acrosomal status. The Q1 region is FITC-PNA−/PI+, representing dead sperm with intact acrosome; Q2 region is FITC-PNA+/PI+, representing dead sperm with acrosome damage; The Q3 region is FITC-PNA+/PI−, representing live sperm with damaged acrosome; Q4 region is FITC-PNA−/PI−, representing live sperm with intact acrosome. (**F**) Sperm viability and acrosome integrity. Data are expressed as means ± SD (n = 3). Different lowercase letters indicate statistically significant differences (*p* < 0.05).

As illustrated in Figure 1A–F and Figure S1, the addition of riboflavin treatment significantly increased the proportion of live sperm with intact acrosome (Q4) (*p* < 0.05). Notably, the percentage of intact viable acrosome sperm in the groups with 5, 10, and 15 μM riboflavin added was significantly better than that in the control group (*p* < 0.05).

### 3.3. Riboflavin Increases MMP and ATP Levels in Sperm

**Figure 2:**
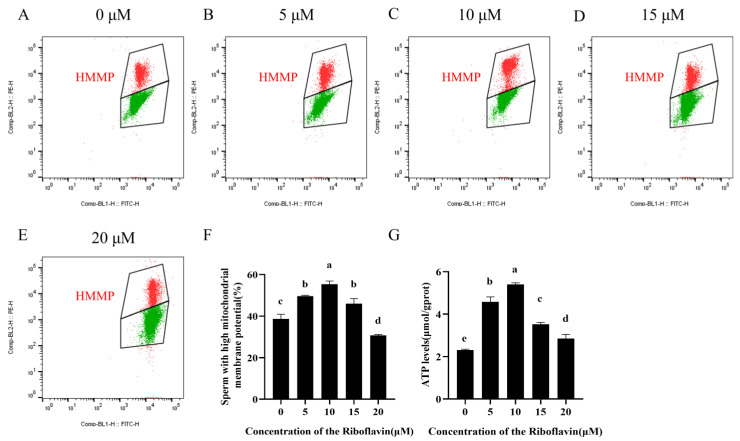
Effects of riboflavin on mitochondrial membrane potential (MMP) and ATP level in goat sperm after 3 h incubation. (**A**–**E**) Flow cytometric analysis of sperm MMP; Red fluorescence indicates sperm with high mitochondrial membrane potential, while green fluorescence indicates sperm with low mitochondrial membrane potential. (**F**) Quantitative comparison of the sperm subpopulation with high MMP. (**G**) ATP levels in sperm. Data are expressed as means ± SD (n = 3). Different lowercase letters denote statistically significant differences (*p* < 0.05).

As demonstrated in Figure 2A–F and Figure S2, riboflavin supplementation significantly improved sperm MMP compared to the control group at all tested concentrations except 20 μM (*p* < 0.05), with the 10 μM riboflavin group showing the highest MMP values. Notably, MMP levels in the 15 and 20 μM riboflavin groups were significantly lower than those in the 10 μM group (*p* < 0.05). Concurrently, Riboflavin supplementation exerted a concentration-dependent effect on ATP levels after incubation, with the highest ATP production observed at 10 μM (*p* < 0.05, Figure 2G). Importantly, higher riboflavin concentrations did not lead to further increases in ATP levels but instead showed a gradual decline.

### 3.4. Riboflavin Optimizes Mitochondrial Energy Metabolism in Goat Sperm

**Figure 3:**
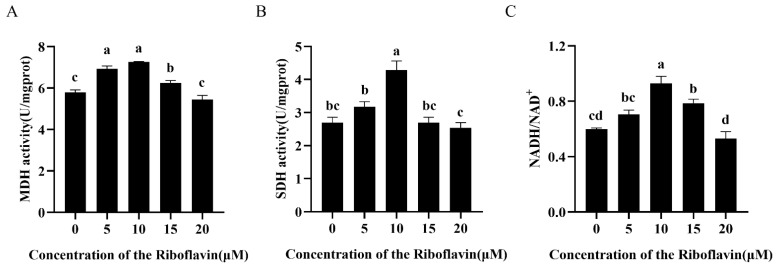
Effect of riboflavin supplementation on sperm MDH (**A**), SDH activity (**B**), and NADH/NAD+ levels (**C**). MDH: malate dehydrogenase; SDH: succinate dehydrogenase. The data were expressed as means ± SD (n = 3), with different letters indicating significant differences (*p* < 0.05).

MDH and SDH are two pivotal enzymes in the TCA cycle. As shown in Figure 3A, incubation with 5 and 10 μM riboflavin significantly enhanced MDH activity compared to the control (*p* < 0.05). Notably, the 10 μM riboflavin group demonstrated the highest SDH activity among all experimental groups (Figure 3B). The NADH/NAD^+^, a key indicator of TCA cycle flux, reflects cellular respiratory status, elevated ratios correlate with heightened OXPHOS. Similarly, riboflavin supplementation (5–15 μM) markedly increased the NADH/NAD^+^ levels, with 10 μM riboflavin achieving the optimal enhancement (Figure 3C, *p* < 0.05 vs. control).

### 3.5. Riboflavin Enhances Mitochondrial β-Oxidation in Goat Sperm

**Figure 4:**
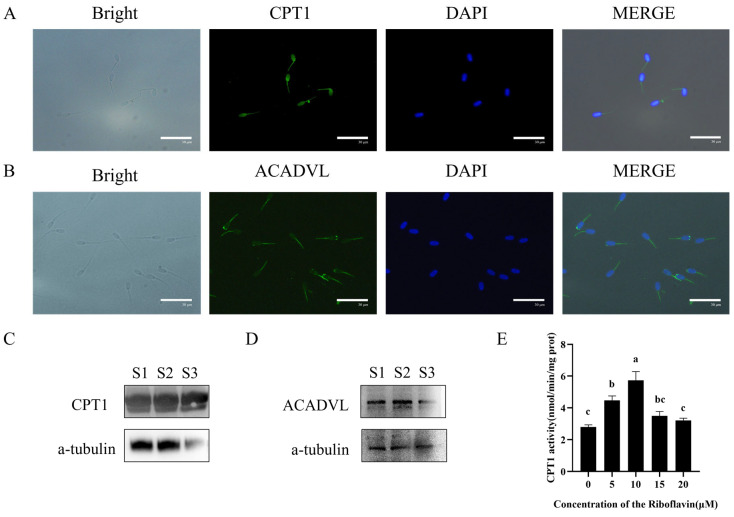
Detection and immunofluorescence localization of CPT1 (**A**) and ACADVL (**B**) in goat sperm. Western blot analysis of CPT1 (**C**) and ACADVL (**D**) in goat sperm. (**E**) Effect of riboflavin supplementation on CPT1 activity. CPT1: carnitine palmitoyltransferase 1. ACADVL: long-chain acyl-coenzyme A dehydrogenase. The data were expressed as means ± SD (n = 3), with different letters indicating significant differences (*p* < 0.05). S1: Goat sperm 1, S2: Goat sperm 2, S3: Goat sperm 3.

To validate the hypothesis that riboflavin maintains sperm motility by promoting ATP production via fatty acid β-oxidation, we first investigated the expression and localization of two key β-oxidation enzymes—CPT1 and ACADVL—in goat sperm using Western blotting and immunofluorescence. CPT1, the rate-limiting enzyme for mitochondrial β-oxidation responsible, exhibits distinct subcellular localization in goat sperm, with prominent expression observed at the sperm head and mitochondrial compartments (Figure 4A,C and Figure S3). ACADVL, the enzyme catalyzing the initial step of fatty acid β-oxidation, was predominantly localized in the sperm midpiece, where mitochondria are densely distributed (Figure 4B,D and Figure S4). Crucially, riboflavin treatment (10 μM) during 3 h incubation significantly elevated CPT1 enzymatic activity compared to controls (*p* < 0.05) (Figure 4E). These findings collectively demonstrate that appropriate amounts of riboflavin can improve sperm quality by enhancing β-oxidation.

### 3.6. Effect of Selective CPT1 Inhibitor Etomoxir on Sperm Motility, CPT1 Activity, and ATP Levels

**Figure 5:**
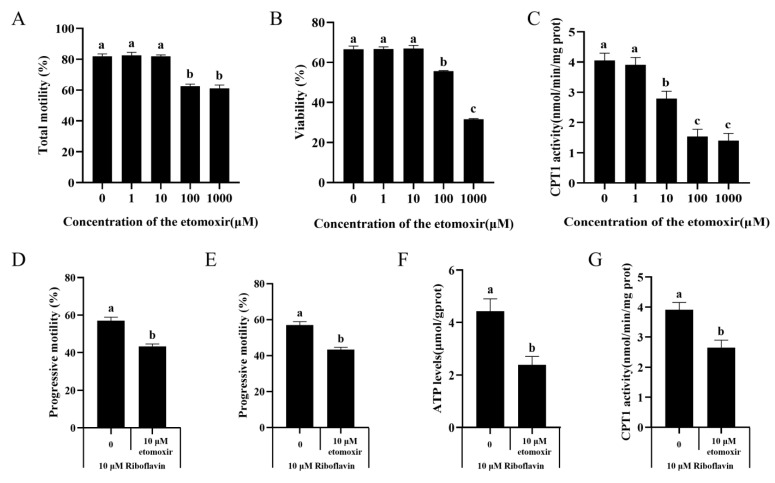
Effects of different concentrations of etomoxir on total sperm motility (**A**), viability (**B**) and CPT1 activity (**C**). Effect of etomoxir added to the medium in the presence of 10 μM riboflavin on total motility (**D**), progressive motility (**E**), ATP levels (**F**) and CPT1 activity (**G**) of sperm. Data are the means ± SD (n = 3). Columns with different lowercase letters differ significantly (*p* < 0.05).

Etomoxir is reported to be a CPT1 inhibitor that can be used to assess β-oxidation of sperm fatty acids. In this study, we treated sperm with etomoxir and observed a significant decrease in total sperm motility at concentrations of 100 and 1000 μM, while other treatment groups showed similar results to the control (Figure 5A). Similarly, under the treatment of 0, 1 and 10 μM etomoxir, there were no significant differences in viability (Figure 5B). Additionally, CPT1 activity decreased at 10, 100, and 1000 μM etomoxir, with the 100 and 1000 μM groups showing significantly lower activity than the others (Figure 5C). Based on these results, we further co-incubated sperm with 10 μM etomoxir and 10 μM riboflavin. The results showed that the addition of etomoxir significantly reduced total sperm total motility (Figure 5D), progressive motility (Figure 5E), CPT1 activity (Figure 5F), and ATP levels (Figure 5G).

## 4. Discussion

In this study, we demonstrated for the first time that CPT1 and ACADVL, two key enzymes involved in mitochondrial β-oxidation, are expressed and localized to caprine sperm. During sperm incubation, riboflavin supplementation exerted a dose-dependent enhancement on sperm motility parameters, with 10 μM riboflavin producing the most pronounced effects. After 3 h of incubation, treatment with 10 μM riboflavin significantly increased sperm motility and acrosome integrity, CPT1 activity, ATP levels, mitochondrial activity, and TCA cycle enzyme activities, whereas higher concentrations did not further enhance these qualities. Notably, the addition of etomoxir, a specific inhibitor of mitochondrial β-oxidation, partially attenuated these riboflavin-induced enhancements. These findings suggest that goat sperm may use fatty acids to maintain viability, and that appropriate riboflavin supplementation promotes mitochondrial β-oxidation and ATP production in a dose-dependent manner, thereby improving sperm quality.

Mitochondria are the central hub of cellular energy metabolism, and its dysfunction has been implicated in a wide spectrum of pathophysiological disorders, including metabolic syndromes and reproductive abnormalities [33]. In the male reproductive system, mitochondrial activity is closely linked to sperm quality, viability, and fertilization capacity [34]. Declines in mitochondrial membrane potential or structural abnormalities in the electron transport chain can lead to excessive reactive oxygen species (ROS) accumulation, disrupting redox homeostasis, impairing motility, inducing oxidative DNA damage, and contributing to male infertility [35]. Therefore, supplementation with nutrients or metabolic cofactors that support mitochondrial function represents a promising strategy to improve sperm performance.

Riboflavin has demonstrated considerable potential in mitigating disorders associated with mitochondrial dysfunction [16,36]. In boar sperm, for instance, supplementation with 10 μM riboflavin during cryopreservation reduced oxidative stress and DNA damage, preserved sperm structure, and decreased apoptosis, whereas excessive riboflavin increased oxidative stress [37]. Consistent with these findings, our study revealed dose-dependent improvements in goat sperm quality, with 10 μM producing optimal benefits. At this concentration, sperm motility, acrosome integrity, and membrane stability were significantly improved, whereas higher doses attenuated these effects, consistent with an inverted U-shaped response commonly observed for vitamins and redox-active cofactors [38]. These findings highlight the importance of optimizing riboflavin dosage to maximize functional benefits. It should be noted that excessively high sperm concentrations may lead to increased competition for nutrients and elevated oxidative stress, which have been associated with genetic toxicity effects, lipid peroxidation damage, and reduced sperm motility. In the present study, these potential confounding effects were minimized by standardizing the sperm concentration across all experimental groups. Nevertheless, future studies are warranted to systematically evaluate the specific influence of sperm concentration on these parameters.

Mitochondria are the primary sites of ATP synthesis, providing the energy required for numerous cellular processes [39]. In sperm, ATP is indispensable for supporting sperm viability, motility, capacitation, and fertilization competence. In this study, incubation with 10 μM riboflavin for 3 h significantly increased MMP and ATP production in goat sperm. One of the principal metabolic pathways responsible for ATP production is OXPHOS, which occurs within the inner mitochondrial membrane. To gain deeper insights into the influence of riboflavin on mitochondrial function, we assessed the activities of two key mitochondrial enzymes: SDH and MDH, both of which play integral roles in the TCA cycle and the electron transport chain. Incubation with 10 μM riboflavin markedly enhanced the catalytic activity of both enzymes, indicating upregulated mitochondrial oxidative metabolism. Consistent with these results, the NADH/NAD^+^ levels were also significantly elevated, reflecting improved mitochondrial redox balance and respiratory efficiency. These findings suggest that riboflavin stimulates mitochondrial activity and ATP production via OXPHOS, thereby modulating sperm energy metabolism.

A growing body of evidence has identified fatty acid oxidation (FAO) as a critical and evolutionarily conserved pathway for energy acquisition in sperm [40]. During this process, fatty acids undergo β-oxidation to yield acetyl-CoA, which subsequently feeds into TCA cycle to generate reducing equivalents for the electron transport chain, thereby driving mitochondrial OXPHOS and sustaining ATP production. Notably, Slc22a14-mediated FAO has been shown to regulate energy metabolism and motility in murine sperm, emphasizing the indispensable role of this pathway in maintaining sperm vitality and fertilizing ability [28]. Therefore, riboflavin may enhance ATP production through OXPHOS and provide additional energy sources by promoting FAO, thereby improving sperm motility. To validate this hypothesis, in this study, we conducted an in-depth analysis of the mitochondrial β-oxidation system in goat sperm and successfully identified the key enzymes related to this process. Among them, we confirmed the expression of CPT1 and ACADVL in goat sperm, and through localization analysis, we found that these two enzymes were mainly distributed in the mitochondrial region of sperm. This demonstrates that goat sperm possesses a complete mitochondrial β-oxidation system, aligning with findings in other species and solidifying FAO’s central role in sperm bioenergetics [32,40]. To further explore the regulatory role of riboflavin in the FAO process, we detected the enzyme activity of CPT1 and found that 10 μM riboflavin significantly enhanced enzyme activity. This suggests that riboflavin can promote the fatty acid oxidation process and improve the energy supply capacity of sperm. Etomoxir is an inhibitor of the specific CPT1 enzyme and can effectively inhibit the activity of CPT1 [41]. Notably, treatment with 10 μM etomoxir partially blocked the beneficial effects of 10 μM riboflavin, leading to reduced CPT1 activity, ATP levels, and sperm motility. These results confirm the central role of FAO in riboflavin-mediated enhancement of sperm vitality.

Overall, this study demonstrates that riboflavin supplementation at appropriate concentrations can stimulate mitochondrial β-oxidation, increase ATP levels, and enhance sperm motility. This trial provides a simple strategy for the screening of effective additives in goat semen, but it is important to note that the effect of riboflavin supplementation during in vitro sperm preservation has not been tested and evaluated, and further studies are needed to assess its effect on sperm performance.

## 5. Conclusions

In conclusion, this study shows that riboflavin at appropriate concentrations can regulate sperm ATP production through multiple energy metabolism pathways, including enhancing the enzyme activity in the OXPHOS process, increasing the NADH/NAD^+^ levels, and promoting the FAO pathway, providing a new perspective and theoretical basis for improving sperm vitality. These findings advance our understanding of sperm energy metabolism and provide a theoretical foundation for optimizing semen preservation and artificial insemination strategies, while emphasizing the critical importance of dose optimization.

## Figures and Tables

**Table 1:** Effect of Riboflavin on goat sperm motility parameters measured with CASA during incubation.

Sperm Parameters	Time (h)	0 µM	5 µM	10 µM	15 µM	20 µM
Total motility (%)	0	82.4 ± 2.7	83.7 ± 4.5	85.5 ± 2.6	85 ± 1.6	84 ± 2.7
	1	80.9 ± 1.5	81.4 ± 2.5	80.5 ± 2	81.3 ± 0.8	82.4 ± 1.5
	3	68.4 ± 1.2 ^b^	76.2 ± 1.4 ^a^	79.3 ± 1.6 ^a^	73.2 ± 5.1 ^a^	75.6 ± 4 ^ab^
	6	65.9 ± 2.8 ^c^	70.8 ± 0.8 ^b^	76.2 ± 1.8 ^a^	67.6 ± 1.9 ^bc^	65.9 ± 2.8 ^c^
Progressive motility (%)	0	71 ± 0.8	71.5 ± 2.3	72.8 ± 1.4	72.8 ± 0.9	71.6 ± 0.9
	1	64.4 ± 2.7 ^b^	68.5 ± 1.4 ^a^	70 ± 0.7 ^a^	69.3 ± 1.7 ^a^	70.2 ± 1.4 ^a^
	3	54.3 ± 2.3 ^c^	64.9 ± 1.4 ^ab^	68.6 ± 0.2 ^a^	62.6 ± 2.3 ^b^	60.6 ± 4.7 ^b^
	6	43.3 ± 1.4 ^c^	47.3 ± 0.9 ^b^	53.3 ± 2.1 ^a^	48 ± 1.9 ^b^	48.5 ± 1.5 ^b^
VCL (µm/s)	0	175.6 ± 5.7	155.1 ± 25.9	163.6 ± 1.3	169.3 ± 4.9	168.4 ± 13.8
	1	152.7 ± 6.6 ^a^	156 ± 2.9 ^a^	136.2 ± 4.2 ^b^	139.5 ± 13 ^b^	135 ± 0.2 ^b^
	3	135.6 ± 10.2 ^b^	164.6 ± 1.7 ^a^	165.8 ± 5.6 ^a^	129.7 ± 5.2 ^b^	145 ± 13.6 ^b^
	6	113.3 ± 5 ^b^	116.8 ± 2.3 ^b^	131.5 ± 5 ^a^	132 ± 5 ^a^	127.3 ± 2.2 ^a^
VSL (µm/s)	0	101.2 ± 6.9 ^a^	86.3 ± 4 ^b^	95.2 ± 1.5 ^ab^	96 ± 6.9 ^a^	95.4 ± 1.9 ^ab^
	1	76.9 ± 3.3 ^ab^	82.2 ± 1.9 ^a^	72.8 ± 2.6 ^b^	75.6 ± 6.4 ^ab^	77.4 ± 2.1 ^ab^
	3	72.5 ± 3 ^b^	91.2 ± 2.9 ^a^	94.6 ± 0.9 ^a^	69 ± 1.5 ^b^	72.5 ± 5.7 ^b^
	6	57.9 ± 1.6 ^b^	61.6 ± 1.5 ^b^	67.6 ± 3.5 ^a^	67.8 ± 1.8 ^a^	67.9 ± 0.3 ^a^
VAP (µm/s)	0	112.1 ± 6.5 ^a^	96.7 ± 5.8 ^b^	105.9 ± 1.4 ^ab^	107.3 ± 5.2 ^a^	106.5 ± 3.8 ^ab^
	1	88.8 ± 3.3 ^ab^	92.4 ± 0.9 ^a^	81.6 ± 2.6 ^b^	85 ± 7.6 ^ab^	85.3 ± 1.6 ^ab^
	3	82 ± 4.7 ^b^	102.3 ± 1.6 ^a^	104.5 ± 0.5 ^a^	78.1 ± 1.5 ^b^	82.6 ± 7.1 ^b^
	6	68.5 ± 2.8 ^b^	74.1 ± 2.4 ^b^	80.5 ± 4.1 ^a^	80.9 ± 3 ^a^	80.1 ± 1.6 ^a^
BCF (Hz)	0	41.2 ± 4.2 ^a^	33.4 ± 0.5 ^b^	38.4 ± 2.6 ^ab^	36.2 ± 1.9 ^ab^	36.9 ± 1.9 ^ab^
	1	31 ± 0.9	34.8 ± 1.2	31.5 ± 2.2	31.6 ± 3.3	33.2 ± 4.7
	3	31 ± 2 ^b^	36.1 ± 1.1 ^a^	38.5 ± 1.3 ^a^	29.5 ± 1.3 ^b^	31 ± 1.4 ^b^
	6	25.5 ± 1.6 ^bc^	24.4 ± 0.5 ^c^	28.8 ± 0.8 ^a^	27.2 ± 0.5 ^ab^	27.1 ± 0.7 ^ab^
ALH (µm)	0	6 ± 0.3	6 ± 0.7	5.7 ± 0.1	6 ± 0.6	6 ± 0.5
	1	6.3 ± 0.3 ^a^	6 ± 0.3 ^ab^	5.5 ± 0.1 ^b^	5.6 ± 0.2 ^b^	5 ± 0.4 ^c^
	3	5.6 ± 0.7	6.1 ± 0.1	5.7 ± 0.3	5.4 ± 0.3	6.1 ± 0.2
	6	5.9 ± 0.1	6.1 ± 0.2	6 ± 0.1	6.2 ± 0.1	6.1 ± 0.2
STR (%)	0	90.2 ± 1.3 ^b^	93.1 ± 0.9 ^a^	89.8 ± 0.2 ^b^	88.6 ± 1.9 ^b^	89.1 ± 1.5 ^b^
	1	85.5 ± 0.7 ^b^	88.2 ± 2.6 ^a^	88.6 ± 0.5 ^a^	88.7 ± 0.6 ^a^	90.4 ± 1.2 ^a^
	3	87.6 ± 2 ^ab^	88.6 ± 1.6 ^ab^	90 ± 0.4 ^a^	88 ± 1 ^ab^	86.9 ± 0.3 ^b^
	6	83.2 ± 1.8	81.4 ± 1.5	82.8 ± 0.2	82.5 ± 0.9	83.1 ± 1.2
LIN (%)	0	58.9 ± 2.3	58.4 ± 4.6	59.4 ± 1	57.4 ± 5.3	57.8 ± 3.3
	1	50.9 ± 0.7 ^c^	53.7 ± 1.8 ^b^	54.2 ± 0.9 ^b^	55.2 ± 1.4 ^a^	58.5 ± 1.7 ^b^
	3	54.1 ± 2.3 ^b^	56.3 ± 1.6 ^ab^	58.2 ± 1.5 ^a^	53.9 ± 2.1 ^b^	50.4 ± 0.5 ^c^
	6	50.6 ± 1.4 ^b^	52.3 ± 1.2 ^ab^	51.2 ± 0.9 ^ab^	51.1 ± 1 ^ab^	52.9 ± 0.3 ^a^

Date is expressed as means ± standard deviation (SD) (n = 3). Different letters in a row indicate significant differences (*p* < 0.05). VCL, curvilinear velocity; VSL, straight-line velocity; VAP, average path velocity; BCF, beat-cross frequency; ALH, lateral head; STR, straightness (VSL/VAP); LIN, linearity (VSL/VCL).

## Data Availability

Upon reasonable request, the datasets of this study can be made available from the corresponding author.

## Supplementary Materials

The following supporting information can be downloaded at: https://www.mdpi.com/article/10.3390/biology15010085/s1, Figure S1: Effect of Riboflavin supplementation at different concentrations on sperm viability and acrosome integrity in goats; Figure S2: Effect of Riboflavin supplementation at different concentrations on mitochondrial membrane potential of goat sperm. Figure S3: The protein expression of CPT1 in goat sperm. Figure S4: The protein expression of ACADVL in goat sperm.
