# Supplementary material for: Riboflavin Increases Goat Sperm Motility via Enhancement of Mitochondrial β-Oxidation
Source: Biology (Basel). 2025 Dec 31;15(1):85. doi: 10.3390/biology15010085 (PMC12784946; doi:10.3390/biology15010085)

Figure S1. Effect of riboflavin supplementation at different concentrations on sperm viability and acrosome integrity in goats.

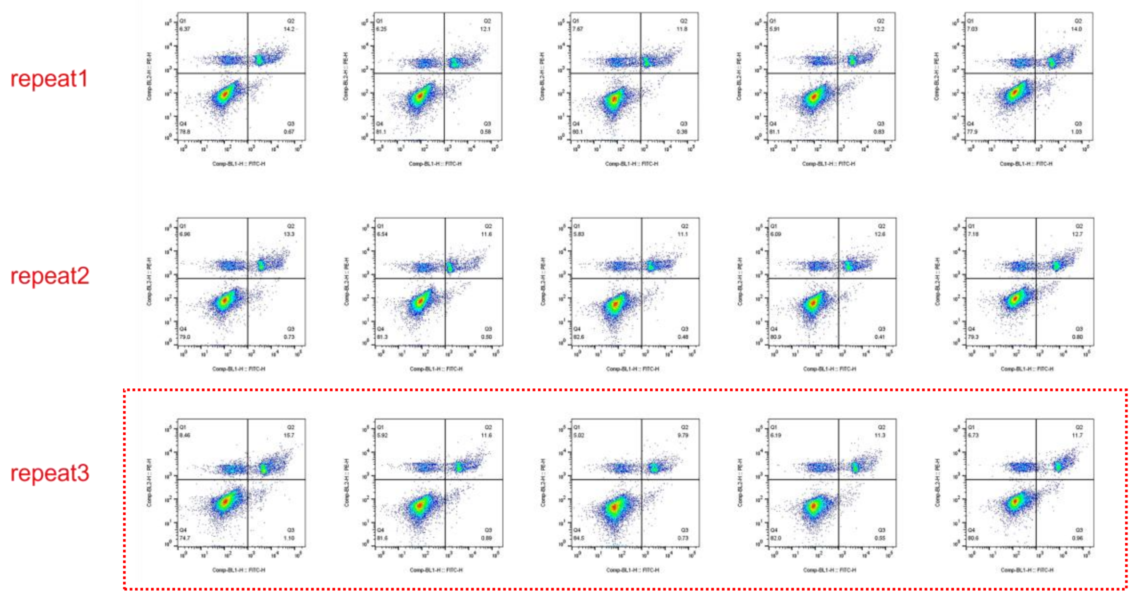

Figure S2. Effect of riboflavin supplementation at different concentrations on mitochondrial membrane potential of goat sperm.

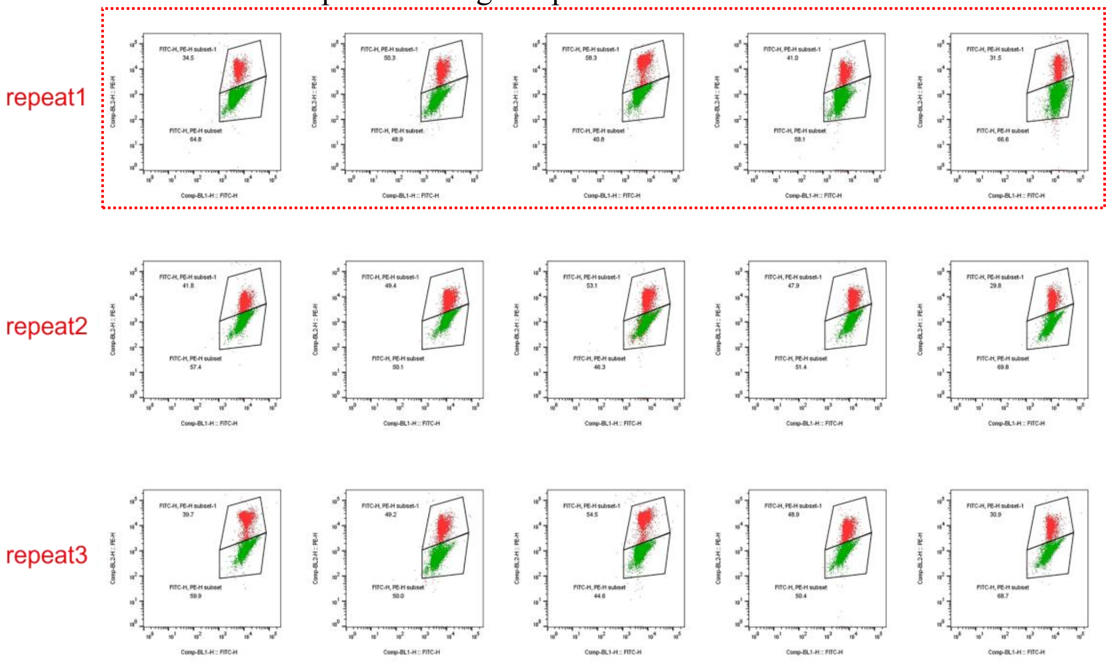

Figure S3. The protein expression of CPT1 in goat sperm.

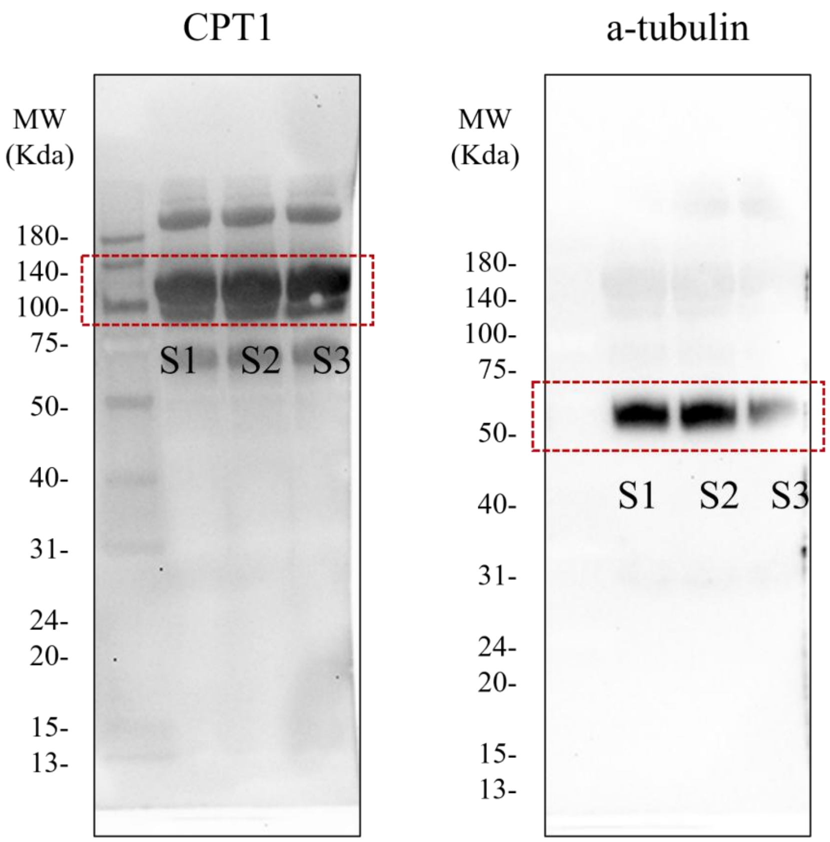

Figure S4. The protein expression of ACADVL in goat sperm.

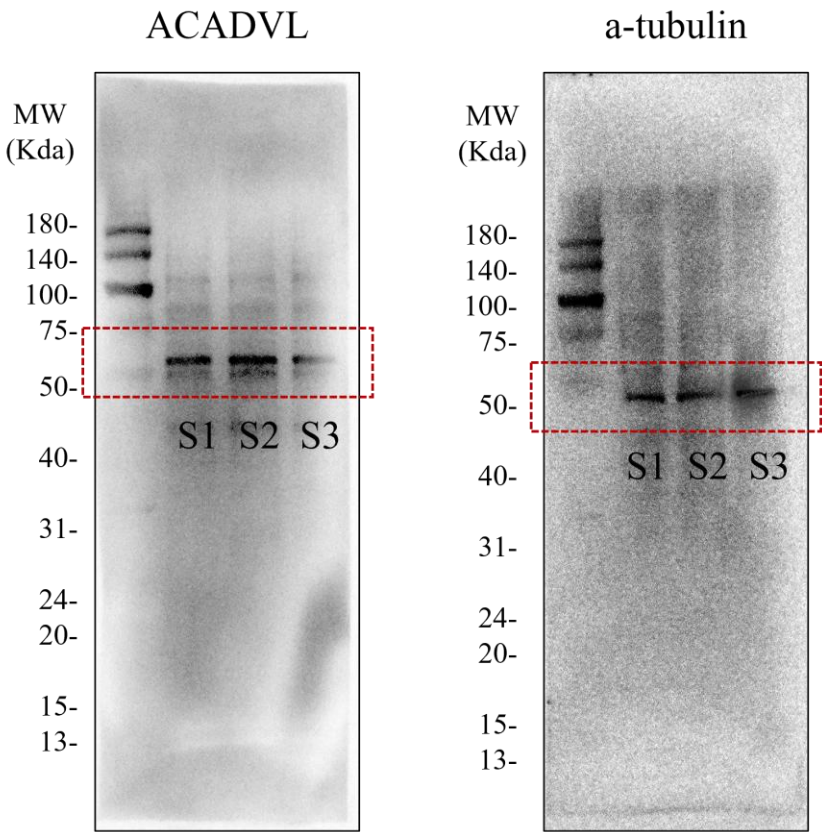

Supplement: Supplementary file 1 [file biology-15-00085-s001.zip › biology-4037853-supplementary.pdf]
